# Supplementary figures and images for: Enteric nervous system modulation of luminal pH modifies the microbial environment to promote intestinal health
Source: PLoS Pathog. 2022 Feb 10;18(2):e1009989. doi: 10.1371/journal.ppat.1009989 (PMC8830661; doi:10.1371/journal.ppat.1009989)

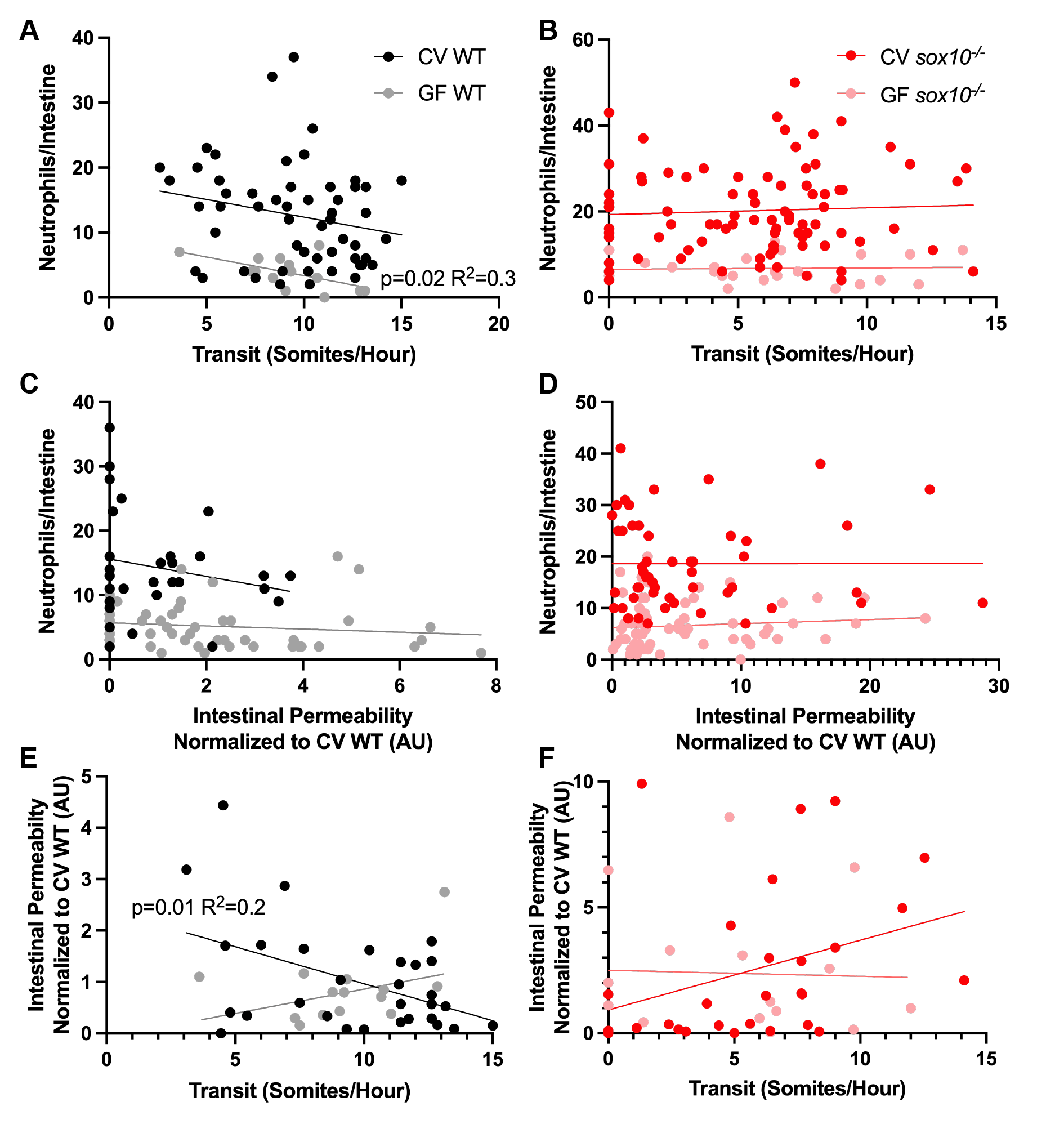

Supplement: S1 Fig — Correlation analysis of (A) WT and (B) sox10 mutant transit and neutrophils. Correlation analysis of (C) WT and (D) sox10 mutant permeability and neutrophils. Correlation analysis of (E) WT and (F) sox10 mutant transit and permeability. Each dot is a fish; n>18 for all conditions. Simple linear regression analysis on CV WT, GF WT, CV sox10, and GF sox10 data points. (TIF) [file ppat.1009989.s001.tif]

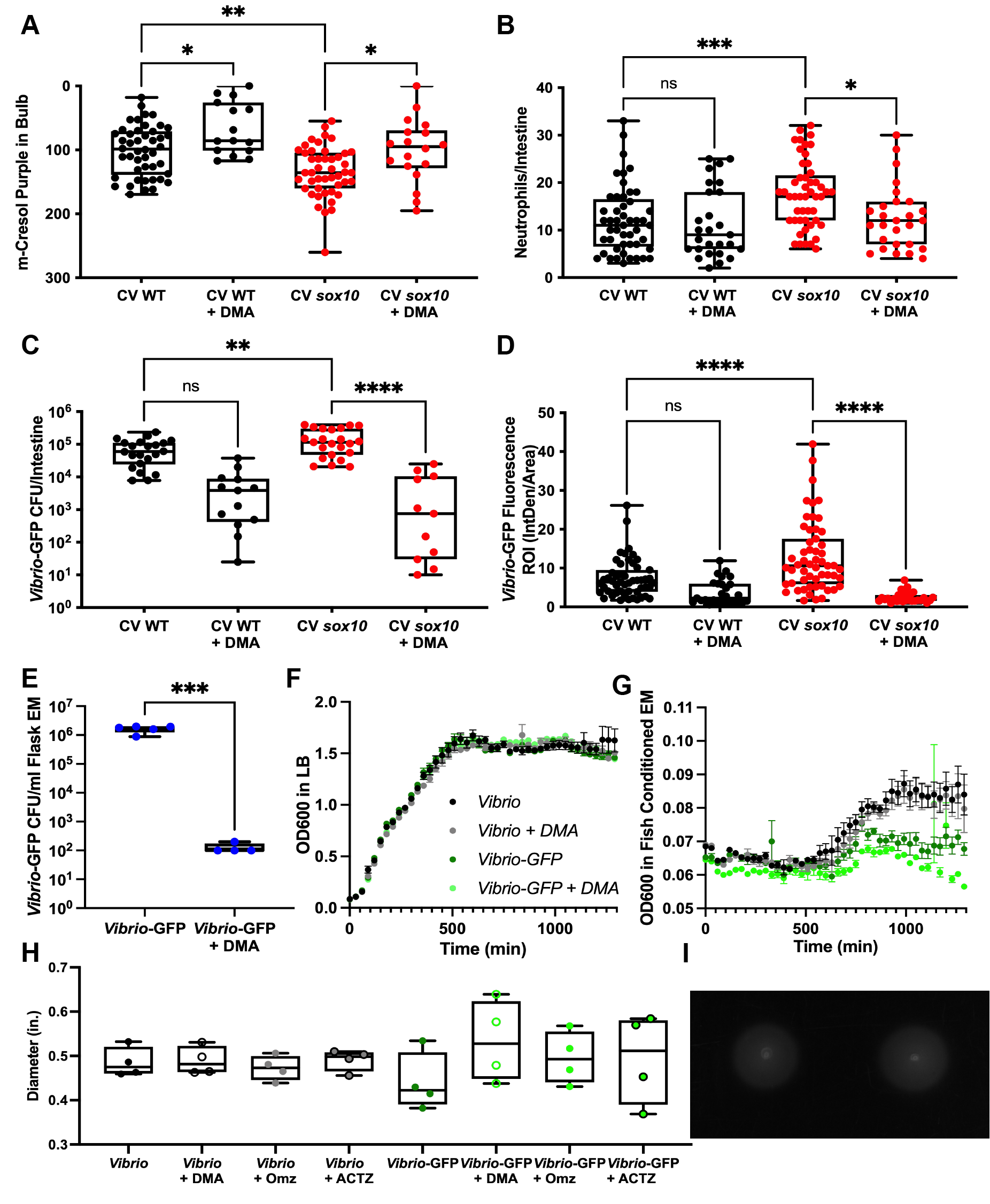

Supplement: S2 Fig — (A) Quantification of luminal pH in intestinal bulb plotting the value of red + green–blue integer values (see Methods) of CV WT and sox10 mutants after 24 hour exposure to 100 μM N,N-Dimethylamiloride (DMA) 20 min after m-Cresol Purple gavage. (B) Quantification of intestinal neutrophil number per distal intestine in CV WT and sox10 mutants after 24 hour exposure to 100 μM DMA. (C) Quantification of Vibrio-GFP colonization level (Colony Forming Units/intestine) in CV WT and sox10 mutants after 24 hour exposure to Vibrio-GFP and 100 μM DMA (CV WT vs CV WT+DMA t-test p = 0.0003). (D) Quantification of Vibrio-GFP luminal fluorescent intensity in CV WT and sox10 mutants after 24 hour exposure to Vibrio-GFP and 100 μM DMA. (E) Quantification of Vibrio-GFP colonization level (CFU/ml) in surrounding EM after 24 hour exposure to 100 μM DMA. (F) Quantification of Vibrio and Vibrio-GFP growth curves in LB and (G) fish conditioned EM with 100 μM DMA. (H) Quantification of Vibrio and Vibrio-GFP swimming diameter in agarose with 100 μM DMA, 200 μM Omz, or 200 μM ACTZ. (I) Representative image of Vibrio and Vibrio+Omz growth diameters in agarose. Each dot is a fish; n>11 (B-D), each dot is a flask (E), each dot is an average absorbance of 4 experimental replicates (F&G). Boxes represent the first to third quartiles, center bar denotes the median, and whiskers the maximum and minimum of each dataset. * p < 0.05, **p<0.01, ***p<0.001, ****p<00001. ANOVA followed by Tukey’s post hoc test. (TIF) [file ppat.1009989.s002.tif]
