# Supplementary material for: Enteric nervous system modulation of luminal pH modifies the microbial environment to promote intestinal health
Source: PLoS Pathog. 2022 Feb 10;18(2):e1009989. doi: 10.1371/journal.ppat.1009989 (PMC8830661; doi:10.1371/journal.ppat.1009989)
Supplement: Attachment — Submitted filename: Response to Reviewers_Hamiltonetal.docx [file ppat.1009989.s005.docx]

*Part I - Summary
Please use this section to discuss strengths/weaknesses of study, novelty/significance, general execution and scholarship.*

*Reviewer #1: Host-microbe interactions are of the utmost importance when it comes to pursuing a holistic understanding of the many factors that affect the health of macroorganisms. In humans and all other animals studied to date, these interactions with microbes are particularly impactful in the gastrointestinal tract. A “healthy” relationship between host and microbiota generally supports host immune function, colonization resistance against invading pathogens, host and microbe nutrition, and many other aspects of host function. When a dysbiotic state is achieved in the GI tract, often roughly defined by a pro-inflammatory shift in the composition or function of the microbiota, the host tends to suffer the consequences in the form of inflammation and a variety of other negative effects. Much work has been done in recent years with the overarching goal of achieving an integrated understanding of host-microbe and microbe-microbe interactions in the gut. While these efforts have uncovered many important interactions between the host and the microbiota, the role of the enteric nervous system in this complex environment has remained a particularly difficult research topic.
In this study, Hamilton et al. investigate the role of the ENS in maintaining homeostasis in the gut by turning to the larval zebrafish model. By harnessing a sox10 mutant, which lacks an ENS and develops pro-inflammatory dysbiosis, the authors were able to leap-frog from their previous observations in this model in order to gain a more mechanistic understanding of how the ENS regulates the microbial composition of the GI tract. The authors find that the lack of an ENS leads to hyperpermeability of the intestinal epithelium, slower gut transit times, and decreased luminal pH. Importantly, these effects of sox10 mutation were found to be independent of the microbiota.
I found this manuscript to be well-written and the experiments to be elegantly done.*

We appreciate the enthusiastic response.

*****

*Reviewer #2: In the manuscript titled “Enteric nervous system modulation of luminal pH modifies the microbial environment to promote intestinal health,” Hamilton et al. characterized the roles of the enteric nervous system (ENS) in intestinal homeostasis. The authors used the sox10 KO line, which was previously shown to lack ENS, to explore the alterations of intestinal physiology in zebrafish. They showed that sox10 deficient fish had altered intestinal transit, intestinal permeability, and luminal pH regulation. They provided evidence to support their claim that these alterations are independent of the presence of the gut microbiota, and therefore these alterations precede and therefore are causal to the dysbiosis and gut inflammation observed in the sox10 mutant. Hamilton et al. further showed that changes in the luminal pH are likely the cause of the overgrowth of proinflammatory Vibrio species in the sox10 mutant. The authors concluded that a primary function of the ENS is to regulate luminal pH, which plays a critical role in shaping the resident microbial community and regulating intestinal inflammation. This manuscript is well written, the hypotheses were clearly stated, and the evidence provided is largely in support of the author’s claims. However, the interpretation of some key experiments is problematic and will need to be revised what was observed experimentally. Please see specific comments below:*

This is helpful feedback. We have restated the interpretation of the key experiments as we discuss below.

*****

*Reviewer #3: In this study, Hamilton et al utilize a zebrafish model lacking an enteric nervous system (ENS) due to a mutation in the sox10 gene to continue to investigate how the ENS influences the intestinal environment to modulate the gut microbiota. The authors had previously found that the ENS alters the composition of the microbiota, neutrophil recruitment in the gut lumen, and overgrowth of pro-inflammatory Vibrio strains. The authors utilize wild-type zebrafish with a conventional microbiota, wild-type germ-free zebrafish, sox10 mutant zebrafish with a conventional microbiota, and germ-zebrafish sox10 mutant zebrafish and report that intestinal transit, intestinal permeability, and luminal pH regulation are aberrant in sox10 mutants independent of the microbiota. Finally, they find that loss of sox10 leads to a drop in luminal pH and overgrowth of proinflammatory Vibrio strains. The data are incremental but interesting, and the findings have potential to have broad implications on the role of sox10 on pH and growth of proinflammatory members of the microbiota. My major criticism is that a lot of the data is variable and some of the differences reported to make major conclusions are not convincing. The authors would strengthen their conclusions if they repeated some of the experiments to increase the significance and confidence in the data. The manuscript would also benefit from improving the clarity of the writing. My comments are outlined below:*

The reviewer raises an interesting point; however, we respectfully disagree that additional replication of experiments would alter our conclusions. As we discuss in our revised manuscript and in our response below, the phenotypic variability across *sox10* mutant individuals is a phenomenon we have previously reported and shown to be correlated with variability in microbiome composition. Specifically, we observe that the phenotypic distribution of *sox10* individuals is broader than but overlapping with the phenotypic distribution of wild type individuals. Additional replication would not be expected to tighten the phenotypic distribution of *sox10*. Such increased phenotypic variability is common in disease of dysbiosis, which are characterized by a loss of constraints on normal microbiome composition.

*****

***Part II – Major Issues: Key Experiments Required for Acceptance*** *Please use this section to detail the key new experiments or modifications of existing experiments that should be absolutely required to validate study conclusions.

Generally, there should be no more than 3 such required experiments or major modifications for a "Major Revision" recommendation. If more than 3 experiments are necessary to validate the study conclusions, then you are encouraged to recommend "Reject".*

*Reviewer #1: I do not believe that this manuscript requires any major revisions.*

*****

*Reviewer #2: Major issues:

1. Fig. 1D: While the sox10 mutant still exhibited decreased transit time compared to the CV WT, there was no difference between GF sox10 and WT, therefore the decrease was not due to host genotype per se but the microbiota status. Thus, the authors claim “decreased transit in sox10 mutants still occurred in the GF state” need to be revised to better reflect experiment results.*

In Figure 1 we present the statistics of an ANOVA and Tukey’s post hoc test which does not show a significant difference (p=0.06) in transit rate between GF WT and GF *sox10*. When investigating the difference in transit rate between these groups via an unpaired Student’s t-test, we indeed do find that GF *sox10* have decreased transit compared to GF WT and thus we feel that our claim is still valid. We have added this statistical analysis in the manuscript line 158 and 939 and have included the p-value on the graph following the ANOVA and Tukey’s post hoc test.

*2. While the FD4 experiment showed that the increased intestinal permeability associated with sox10 was not microbiota dependent, the TD4 results suggested increased intestinal permeability was associated with microbiota status (Fig. 1E&F). Given the noted variability of this phenotype in the sox10 mutant, was the discrepancy a result of undersampling, as the FD4 experiment clearly had more data points than the TD4 experiment?*

Collectively we assayed intestinal permeability in more than 10 independent trials utilizing hundreds of fish, so we do not believe that the variability of permeability in the *sox10* mutant is a result of under sampling. We performed the permeability experiments with TD4 as a validation of the FD4 experiment results, testing the same parameter but using a different fluorescent probe and fluorescent channel. In a careful reexamination of our results, we discovered that in our original analysis of the TD4 data, we performed a slightly different normalization than for the FD4 data by normalized values to all CV WT samples, rather than normalizing to CV WT within each experiment. The revision has corrected this. We had also originally reported the statistical significance via an ANOVA and Tukey’s post hoc test; however, upon further consideration of the data, which are not normally distributed and are bounded by 0 (there cannot be negative permeability values) we realized that it would be more appropriate to use the nonparametric Kolmogorov-Smirnov t-test. After these changes in our data analysis, the TD4 results are consistent with the FD4 results in suggesting that increased intestinal permeability is seen in *sox10* mutants even in the absence of microbiota.

*3. While it is plausible that the transmission of microbes was responsible for the comparable neutrophil numbers between WT and the sox10 mutant (Fig 2A), this claim can be better substantiated by doing similar experiments in separately housed animals.*

Figure 2A shows that neutrophil number is significantly higher in *sox10* mutants compared to their WT co-housed siblings at 6 day post fertilization (dpf), but not 4 or 5 dpf. On line 208 we state, “The number of neutrophils in WT increased to levels comparable to co-housed *sox10* mutant siblings by 7 dpf (Fig 2A), which could be due to increased levels of transmission of proinflammatory microbes from dysbiotic *sox10* individuals to WTs.” We agree that to test this hypothesis, one could test the difference in intestinal neutrophil levels in WT siblings reared in isolation or together with *sox10* mutants. Although we agree that this would be an interesting line of investigation, we do not think it is necessary for the conclusion of these experiments: which is that intestinal permeability is significantly increased in *sox10* mutants at 5dpf, preceding the significant increase in neutrophil numbers.

*4. While the authors showed good evidence that luminal pH correlated with Vibrio abundance, it is unclear whether luminal pH directly regulates Vibrio abundance or indirectly through influencing other competing microbes in the microbiota, or the combination of the two. This hypothesis can be easily tested using GF animals mon-colonized with Z20.*

We thank the reviewer for this suggestion. We have now performed this experiment and the results are included in Figure 6E. We show that in mono-associations, *Vibrio* Z20 responds to pH-altering pharmacological treatments in the same manner as in the presence of a CV microbiota. These new data indicate that intestinal pH directly regulates *Vibrio* abundance.

*****

*Reviewer #3: Major issues*

*1. One major concern is in regard to the conclusions based on the statistical differences reported in some of the figures. The author’s use statistical analysis to determine whether phenotypes are dependent on sox10 or the microbiota. However, using statistics alone to make conclusions can be misleading especially when there is there is a quite a bit of variability and overlap in the data between groups. It’s important to also reply on common sense and judgement when interpreting the data and making conclusions. In some cases, the differences don’t appear very different but the authors report that they are statistically different, and in some cases, the data appear different between groups but the authors report that they are not statistically different. For example, in Figure 1D, the authors report that the difference in Transit between GF WT and GF sox10 -/- are not statistically different but it’s clear that there is a clear trend for reduced transit time in GF sox10 -/-. In fact, in GF sox10 -/- six animals had a transit time below the limit of detection, while GF WT had zero animals with this phenotype. In contrast, in Figure 1E the authors report that there is a statistically different (**p<0.01) difference in intestinal permeability between GF WT and GF sox10 -/- although there is also a clear trend, the difference doesn’t appear very different between these groups, with exception to the handful of GF sox10 -/- animals with high intestinal permeability. It’s not clear why in Figure 1E, the difference between GF WT and GF sox10 -/- is significantly different, but not in Figure 1D, especially when the difference between the means actually appears greater in Figure 1D than in Figure 1E. Similar trends are observed in Figure 1C, Figure 1F, Figure 2A, Figure 2B…Statistical analysis is impacted by sample size and it raises concerns about whether additional animals in certain groups would change the statistical analysis and thus the central conclusions of this study. Phenotypes in animal models are complex but given that these reported differences are central to the conclusions of the study, this is an important issue that should be addressed by the authors. In lines 178-179, the author’s acknowledge, “This level of variation in hyperpermeability is similar to the variation in inflammation we previously described in CV sox10 mutants.” However, the fact the variation was “previously described” doesn’t satisfy the discrepancy of the observed vs reported statistical differences in the data.*

We agree that evaluation of data should incorporate statistical analysis along with common sense and judgment. As we now discuss more explicitly in the introduction (line 80 and 129), diseases associated with dysbiosis are characterized by greater variability in microbiome composition and associated phenotypes than seen in healthy controls. Thus, it is not reasonable to expect a simple bimodal distribution of phenotypic states separating WT siblings from mutant individuals. Instead, as we reported previously (Rolig et al., 2017) and we show here, *sox10* mutants are characterized by a greater variance of phenotypes than WT. For example, as the reviewer notes, we observe many *sox10* individuals with intestinal permeability in the WT range, but more mutants with greater intestinal permeability values. The breadth of this phenotypic distribution is apparent because we include such large numbers of individuals in our studies and replicate our experiments extensively. In Rolig et al., 2017, we showed that there was a greater variance in microbiome composition in *sox10* individuals, with the extent of *Vibrio* expansion being correlated with the severity of hyperinflammation. Given what we know about the variability of dysbiotic *sox10* mutants, it does not make sense to expect that further replication would narrow the broad phenotypic distributions of the *sox10* animals and better separate them from WT. As much as possible in our study, we have employed independent measures of phenotypic traits to confirm differences between *sox10* mutants and WT siblings, such as using two different dyes to measure intestinal permeability and pH. As discussed in response to Reviewer 2 above, we have revisited the statistical tests we employed, and in the case of the permeability data we adopted a nonparametric test that is more appropriate for the non-normally distributed data that is bounded by 0. We have expanded our statistical approaches section in the Methods to better clarify why we chose specific statistical tests. To clarify the results seen in Fig 1 D and F, we have added further statistical tests in the manuscript, line 158 and 939, and line 186 and 945 respectively.

*2. The authors should report the P-values for all data and the means in the box and whisker plots.*

This is a helpful suggestion. For clarity, we have chosen to display significance via * p < 0.05, **p<0.01, ****p<00001. In addition, we have now added a supplemental table containing all the p-values for each graph presented along with information about which statistical test was performed.

*3. The authors should repeat experiments with GF WT and GF sox10 -/- in Figure 1C, Figure 1D, Figure 1E, Figure 1F, and others (also see comments below) using additional independent animal groups to demonstrate reproductivity of the reported data. If the data are reproducible, the differences become clearer and the data should remain statistically significant, which will strengthen the conclusions of this study.*

As discussed in response to comment 1 above, we do not expect additional replication of these experiments to make the differences between *sox10* and WT populations more distinct. Instead, our extensive replication of trait measurements across hundreds of fish demonstrates that the *sox10* phenotypes are broadly distributed across a range that overlaps with the WT phenotypes. These phenotypic measurements are consistent with our microbiome analyses reported in Rolig et al., 2017 showing that *sox10* individuals have a broad range of microbiome states, with variable expansions of *Vibrio* species abundance.

4. *In lines 212-215, the author’s state, “The observation that hyperpermeability in sox10 mutants is independent of microbiota and preceded intestinal neutrophil influx by at least a day strongly suggests that ENS-mediated regulation of permeability is required to prevent microbial dysbiosis and intestinal inflammation.” I’m not fully convinced by the 5 dpf differences in Fig. 2A and Fig. 2B. Specifically, the authors report that Fig. 2A is not significant and Fig. 2B is significant, although they both appear to be trending in the same direction and thus the sentence, “…strongly suggests that ENS-mediated regulation of permeability is required to prevent microbial dysbiosis and intestinal inflammation” is an overstatement. The authors should repeat the experiments in Fig. 2A and Fig. 2B to better support their conclusions.*

We agree with the reviewer that results are trending even though they do not achieve significance using a 2-way ANOVA and Šídák multiple comparisons test. We have therefore removed the word “strongly” from line 212 and have corrected the statical approach in the figure legend, line 960.

5. *In Figure 4, the data suggest that the microbiota can also affect intestinal pH. In Figure 5 and Figure 6, the authors should repeat the experiments with omz and ACTZ using GF zebrafish to determine the contribution of the microbiota on inflammation and overgrowth of Vibrio strains (i.e monocolonized with Vibrio).*

We thank the reviewer for this suggestion. We have now performed this experiment and the results are included in Figure 6E. We show that in mono-associations, *Vibrio* Z20 responds to pH-altering pharmacological treatments in the same manner as in the presence of a CV microbiota.

*6. In Fig. 6C, why is the CFU data for CV WT + ACTZ not significantly higher but it is for GFP fluorescence in Fig. 6D? Since GFP fluorescence can be influenced by external factors such as oxygen, which can change due to permeability and inflammation, I would not trust GFP data alone without validation by CFU plating. The authors should address this discrepancy.*

We appreciate the suggestion that we address the discrepancy of statistics when comparing *Vibrio* abundance via dilution plating and fluorescence intestines. As Figure 6B shows, the relationship between measurements of *Vibrio* abundance by fluorescence and by dilution plating is not strictly linear because each assay has different dynamic ranges. We use both measures to conclude that ACTZ treatment increases *Vibrio* abundance in CV WT larvae. We have clarified that even though there is a non-significant increase in *Vibrio* abundance via dilution plating when ACTZ is added to CV WT when tested with an ANOVA and Tukey’s post hoc test, it is significantly different when a Student’s t-test is used. We have added a statement about this in the manuscript at line 340 and 1009.

*7. In Fig. 6C and Fig. 6D, the authors compare CV WT to CV WT + OMZ and also compare CV sox10-/- to CV sox10-/- + OMZ and despite an observable drop in CFU in the CV WT + OMZ group, it’s not clear whether the figure is mislabeled or the authors made a mistake in the writing. The authors should perform a statistical analysis to compare the CV WT + OMZ and the CV sox10-/- + OMZ to determine if there is a difference between those two groups.*

With OMZ, *Vibrio* abundance does indeed decrease significantly in CV WT larvae via a Student’s t-test (p=0.01). This test has been included in the manuscript on line 329. There is no significant difference between CV WT + OMZ and CV *sox10^-/-^* + OMZ as seen on the supplemental statistics page.
*****

**Part III – Minor Issues: Editorial and Data Presentation Modifications**
Please use this section for editorial suggestions as well as relatively minor modifications of existing data that would enhance clarity.

*Reviewer #1: Minor issues:*

*1. I believe the authors would be mistaken to not investigate the possible relationship between intestinal pH disregulation and the other sox10 phenotypes described in the manuscript. It seems to me that while this would require additional experiments, it would not require any methodology that is not already present in the study.*

*The questions I would be happy to see answered are as follows:
a. If luminal pH in sox10 mutants is corrected to WT levels by omeprazole, does that have an effect on gut transit time or hyperpermeability?*

***b.*** *if luminal pH is decreased in WT animals by ACTZ, does that have an effect on gut transit time or hyperpermeability?
c. I would like to stress that I don’t see these experiments as absolutely necessary, but I do think they have a potentially high payoff and would only strengthen the paper.*

We agree with the reviewer that the suggested experiments would be interesting next steps for investigating the mechanistic basis for physiological defects in *sox10* deficient animals. However, we feel that these experiments are outside of the scope of the current study and unnecessary to conclude that pH is the primary driver of dysbiosis in *sox10* mutants.

*2. a. In figure panels 6H and 6I, the authors point is to show that Vibrio benefits from a lower pH. In my opinion, this is the least convincing point of the paper. My thinking is heavily influenced by the pH readouts used in figure 5. While m-cresol purple appears to give a pH value near 10 for WT intestinal pH, phenol red appears to give a value slightly below 9. The fact that the two dyes do not agree (the CV sox10 values appear to be shifted similarly) suggests that these readouts might only be trustworthy as relative pH values, and not necessarily as absolute values (they are never regarded as absolute values in the manuscript, to be fair). The reason I find this important is that panels 6H and 6I show that Vibrio grows well at pH values as high as 9 and even reasonably well at pH 10. It does not read as a particularly solid mechanism for how Vibrio is able to bloom. Additionally, in vitro conditions are notoriously limited in their ability to provide insight into in vivo phenotypes. How exactly the authors should address this point is unclear to me, but I can offer some rough suggestions.*

The reviewer makes valid points about the limitations of the pH analysis in Figure 5. We now clarify in the text (line 261 and 270) that we cannot infer precise pH values in the intestine, based on established colorimetric standards, because the color measurements are distorted when visualized through the larval body wall. Instead, we can use these two pH indicators to make inferences about relative pH changes between treatment groups and broad pH ranges. We agree that the *in vitro* experiments have limitations. To better compare across treatments, we have analyzed our growth curve data further and have provided an additional growth curve metric of the area under the curves, which corroborates that *Vibrio* grows better in lower pH conditions in both LB and EM (Figure 6I and 6J).

*b. If the authors were to show similar curves for other zebrafish commensals that are present at this larval stage, it could help strengthen the argument, assuming that those commensals are negatively affected by lower pH.*

We agree with the reviewer that it would be interesting to survey pH sensitivities of other zebrafish commensals. However, we also acknowledge (as noted in the previous comment) the limited utility of extrapolating such *in vitro* experiments to *in vivo* conditions. A comprehensive analysis of the pH responses of other zebrafish commensals, with validation *in vivo,* would be outside the scope of the current study.

*c. A potentially more powerful approach would be to assess the microbial composition of the GI tract in the WT and sox10 backgrounds with and without omeprazole or ACTZ treatment and, if possible, with and without the presence of the pro-inflammatory Vibrio(s). While this approach would not address the mechanism of Vibrio expansion directly, it would serve to show how the treatments affect microbial composition, which may help to elucidate relationships between Vibrio and other commensals under these conditions.*

Although these would be interesting experiments, we feel that they are outside the scope of the current study.

*Another angle of approach that may be useful is looking at the anti-inflammatory bacteria used in a previous sox10 study (Rolig et al., 2017, PLOS Biology, reference #9 in this manuscript). In that study, E. coli and Shewanella isolates were used to decrease inflammation in CV sox10 mutants. Does addition of these isolates correct the luminal pH or is their anti-inflammatory effect mediated by some other mechanism?*

These are interesting suggestions. We are continuing to investigate the anti-inflammatory mechanisms of *E. coli* and *Shewanella* and our results are consistent with these bacteria reducing intestinal inflammation through mechanisms distinct from altering luminal pH. These ongoing studies are outside of the scope of the present manuscript.

*3. In lines 493-495, the authors argue that the impaired intestinal permeability of sox10 mutants could be an underlying mechanism of the luminal pH decrease. Is this not in opposition to the observation that omeprazole corrects the pH back to WT levels? This would suggest that the pH change is an active process mediated by proton pumps, not a passive process mediated by solute leakage from the host.*

The reviewer raises an interesting point. In our study, we use omeprazole as a blunt treatment to drastically change the luminal pH by blocking proton pumps, but this does not rule out the possibility that passive solute leakage or other proton pump-independent mechanisms contribute to increased acidity in the *sox10* intestine. We discuss several of these possibility mechanisms the discussion (line 434).

*4. a. My last point contains a number of related observations about the work. I find it to be an exciting possibility that the ENS regulates the pH of the gastrointestinal tract and I believe that there could be significant implications if this is true in mammals, especially humans. The manuscript does a good job of leading the reader to understand what some potential implications of these findings are, but I think it is lacking in fair comparison of host physiologies. It appears to me that the GI tract of larval zebrafish is quite different from that of a human. Seeing as this study is at least in part funded by the NIH, I would like to see more discussion throughout the text of how this study relates to the human (or even just mammalian) GI tract. For instance, the proton pump ATP4A is responsible for gastric acidity in humans and is not expressed in the GI tract outside of the stomach, as far as I am aware. How does this compare the localization of proton pumps in larval zebrafish?*

We appreciate the reviewer’s suggestion that we provide more information about the similarities between the zebrafish intestinal tract and the gastrointestinal tracts of mammals. We have addressed this point more throughout the paper, specifically for example, starting at line 285. Interestingly, the proton pump ATP4A is not present in the zebrafish, nor in other agastric fish species, however other proton pump genes are expressed in zebrafish enterocytes. We have included the names of these proton pump genes and added references to analyses of a searchable zebrafish single cell atlas that shows these genes to be expressed in enterocytes.

*b. The vast majority of microbes that inhabit the human host reside specifically within the large intestine, where they can reach loads as high as 1011-13 CFUs/g contents. In the larvae studied here, it appears that the highest microbial burden is carried in the bulb. What are the implications of that? In figure 3, tight junctions are found to impaired specifically in the colon-like region but the implication of that is not discussed. There are more specific instances of physiological differences that could be mentioned but my intention is not for the manuscript to be a defense of zebrafish as a model. What I would like to see is a greater effort to address the relatively large differences between the larval zebrafish GI tract and the mammalian GI tract, with special considerations made for spatial (stomach, small intestine, large intestine, etc.) and environmental differences.*

Our lab has done extensive research regarding the localization of the microbiota within the intestine. *Vibrio* does indeed primarily reside in the bulb, yet, like all intestinal microbes, is continually flushed through the length of the intestine and excreted. We speculate that proinflammatory *Vibrio* products may be sensed most acutely in the more permeable *sox10* distal intestine. We have now discussed these points about *Vibrio* biogeography in line 319 and line 414.

*5. Finally, I would like to thank the authors for this work. It was a clean and enjoyable read.*

We appreciate the reviewer’s enthusiasm.

*****

*Reviewer #2:*

*1. What is EM (line 365)?*

Embryo medium (EM), the environment in which zebrafish larvae are grown. We defined it on line 316. We have clarified line 417 and 452.

*2. While occludin binding to ZO1 was decreased in GF sox10 compared to CV WT, it is unclear (statistics not showed) whether there was a statistically significant difference between CV WT and GF WT, or between GF WT and GF sox10 (Fig. 3A). Therefore, it is unclear whether the hyperpermeability phenotype is independent of the microbiota status, at least in the ileum-like region.*

We did not include statistical information here because there was no significant difference between CV WT and GF WT or between GF WT and GF *sox10*. There was a significant difference between CV WT and GF *sox10* which we have now included for completeness. We have clarified our conclusions of these data in line 269 stating that “tight junction integrity in the ileum-like region is independent of genotype or microbiota, yet tight junction integrity in the colon-like region is dependent on genotype, but independent microbiota.”

*3. To substantiate the claim that “reduced luminal pH of sox10 mutants induces the hyperinflammatory state”, the authors should add the statistics of the comparison of WT and sox10+omz, which will show whether reducing luminal pH is sufficient to reverse the phenotype.*

To address this point, we have now included a supplemental file that contains all the p-values for each condition pair. We have also simplified our graphs by only showing statical differences on our figures. The pH of WT and *sox10*+OMZ was not significantly different, thus we have not put significant values on the graph. The p-value for this pair was p=0.9967, indicating that reducing luminal pH was sufficient to reverse the phenotype, line 298.

*****

*Reviewer #3:*

*1.* In lines 64-65, the authors say, “Using zebrafish lacking an ENS due to a homozygous null mutation in the sox10 gene we discovered that the ENS constrains intestinal microbiota composition and intestinal inflammation, as measured by intestinal neutrophil infiltration.” What is sox10? What it’s function? The authors should add a sentence or two to better introduce sox10. This is important given that sox10 is central to the study.

We appreciate the suggestion. We have added a more thorough introduction of *sox10* at line 64.

*2. In lines 182-183, the authors write, “We observed that even in the absence of microbiota, sox10 mutants had significant levels of fluorescence in the aorta compared to GF WTs (Fig 1E and F).” What does “significant levels” mean? Significant higher or lower levels? The authors call out Fig. 1E and Fig. 1F but in Fig. 1F the GF data is not significant. The authors needs to explain this discrepancy and make the writing more clear.*

We have clarified our meaning in the revised text on line 187.

*3. In lines 174-176, the writing is confusing. The authors state, “CV WTs did not have detectable fluorescence in the vasculature. In contrast, CV sox10 mutants had measurable levels of fluorescence in the aorta compared to CV WTs (Fig 1E and F).” When the authors state, “CV WTs did not have detectable fluorescence in the vasculature.” What figure is that referring to? If they are referring to Fig 1E and F, then what are the data points shown in these figures for CV WT? Presumably, if there are data points for CV WT, then the fluorescence was measurable, correct? The authors need to be more clear about exactly which Figures they are calling out in the writing.*

The reviewer raises a good point. We have now clarified (line 179) that the fluorescence in the WT vasculature was not detectable by eye, but it was visible in *sox10* mutants. Even though we did not see fluorescence in WT larvae, the data points presented in Fig1E and F were the intensity values that the sensitive camera on the microscope could detect.

*4. In Figure 4B and D, the authors should also perform as statistical analysis to compare the CV WT and GF WT and CV sox10-/- group to the GF sox10-/- group to determine the contribution of the microbiota in these groups.*

To clarify all the statistics, we have now included a supplemental file that includes all the p-values for each combination presented. We have also decided to only display significant differences, thus for Figure 4B and D, the comparison between CV and GF are not shown because they are not significantly different. On line 271, we address the potential that the microbiota may influence the luminal pH, however using our pH indicators, we did not detect a significant difference.

*5. In lines 315-316, the author’s write “We hypothesized that the hyperinflammation seen in sox10 mutant intestines could be corrected by increasing the luminal pH to levels similar to WTs.” This sentence, needs more context Why did you hypothesize that?*

Based on the reviewer’s comment, we have reworded this section to better convey our thinking (line 289).

*6. In lines 316-317, the authors wrote, “We first verified that zebrafish intestinal cells have proton pumps using single cell RNA-seq data from intestinal tissue [58].” This sentence is confusing because it reads like the authors did an experiment for this study (which is not shown) but they are citing another study. It seems more appropriate for the sentence to read, “We have found that zebrafish intestinal cells have proton pumps using single cell RNA-seq data from intestinal tissue [58].*

We have reworded this section (line 286) based on the reviewer’s comment.

*7. In line 363-365, the authors state “Omeprazole reduced Vibrio-GFP abundance in both CV WTs and sox10 mutants as shown by GFP-CFU (Fig 6C) and fluorescence (Fig 6D)” and in lines 367-368, “DMA treatment decreased intestinal abundance of Vibrio-GFP (S2 Figs C and D)” However, the data shown in Figure 6C,D and Figure S2C indicates that there is no statistical difference in Vibrio CFU/intestine between CV WT and CV WT + OMZ and CV WT and CV WT + DMA, respectively. It seems unlikely they would have mislabeled both of the figures. There appears to be a large difference in CFU (close to one log) between these groups. The authors should explain this discrepancy.*

We have included Student’s t-test p-values on line 329 and 334. We chose to present the statistics from the Tuckey post hoc test, which shows that the variability between groups even though the points show a marked decrease in CFU values with Omz or DMA treatment, it was not significant. To see the p-values after the ANOVA statistical test, we have included an excel spread sheet with all the p-values of each condition combination.

*8. Since DMA has antibacterial activity in vitro independent of zebrafish, it’s not clear why this data is included in this study since it’s not clear whether the in vivo data are due to acidity other to direct antibacterial activity of DMA.*

The reviewer raises a question about inclusion of the DMA data. To clarify, we chose to include these data as supplementary information because although we cannot conclude whether DMA decreases *Vibrio* abundance through increasing luminal pH, the experiment is still instructive. Regardless of the mechanism by which DMA decreases *Vibrio* abundance, the pharmacological treatment provides independent support that decreasing *Vibrio* abundance in *sox10* mutants resulted in decreased neutrophil abundance.
